# F234. TYPICAL AND ATYPICAL ANTIPSYCHOTICS’ D2R AFFINITY AND DOSES INFLUENCES POSTSYNAPTIC DENSITY BY MODULATING THE SPATIAL EXPRESSION OF HOMER1A A GENE HIGHLY IMPLICATED IN SYNAPTIC PLASTICITY AND PSYCHOSIS

**DOI:** 10.1093/schbul/sby017.765

**Published:** 2018-04-01

**Authors:** Avagliano Camilla, Elisabetta Buonaguro, Carmine Tomasetti, Federica Marmo, Licia Vellucci, Felice Iasevoli, Andrea de Bartolomeis

**Affiliations:** University School of Medicine Federico II

## Abstract

**Background:**

Post-synaptic density (PSD) is an ultra-specialized structure of excitatory synapses composed by a large variety of molecules (scaffolding proteins, glutamate receptors, cytoskeleton proteins). PSD has been implicated in synaptic plasticity, memory formation and in the pathophysiology of psychiatric disorders by extensive GWA studies. The immediate early gene Homer1a is part of this complex molecular machinery for signaling transmission and its expression is modulated by antipsychotics (APDs). Here we show a comparative analysis of Homer1a expression data by first and second-generation APDs, in order to correlate it to their receptor profile.

**Methods:**

We analyzed Homer1a expression induced by APDs at various doses in Sprague-Dawley rat forebrain, collecting data from multiple In Situ Hybridization experiments carried out in our laboratory in standard controlled conditions. Homer1a expression levels were normalized as the ratio of the corresponding mean vehicle value in each region. Normalized expression levels were quantitatively compared by ANOVA and Tukey’s post-hoc test (p<.05) and grouped in four classes: no induction; light induction; moderate induction; high induction.

**Results:**

In the striatum, sertindole did not induce Homer1a expression. Quetiapine and amisulpride were observed to trigger light induction of the gene. Clozapine triggered a light-moderate induction. Moderate induction was found by olanzapine and aripiprazole, while high induction was found by ziprasidone, asenapine, and haloperidol, especially in caudate-putamen regions.

In the cortex, Homer1a mRNA was not induced by sertindole, 4mg/kg ziprasidone, haloperidol (0.25 and 0.5mg/kg). Haloperidol 0.8mg/kg, 15mg/kg quetiapine, 10mg/kg and 35mg/kg amisulpride triggered light induction. Moderate induction was found for 30mg/kg quetiapine, olanzapine, clozapine, 10mg/kg ziprasidone and for asenapine at all doses tested. Notably, both clozapine and 10mg/kg ziprasidone induced the highest levels of Homer1a mRNA in the insular cortex.

**Discussion:**

A strong correlation with D2 receptor blockade and the extent of Homer1a expression in striatum, but not in the cortex, was found. However, other molecular mechanisms (e.g. D1 receptor activation in striatum; 5-HT2A receptor blockade in the cortex) may contribute to affect its expression levels.

